# Composition of fish egg assemblages varies with depth on the West Florida Shelf

**DOI:** 10.7717/peerj.20498

**Published:** 2026-01-22

**Authors:** Arianna Rodriguez, Keith Keel, Glenn Zapfe, Kaili Qiao, Yonggang Liu, Christopher D. Stallings, Mya Breitbart

**Affiliations:** 1 College of Marine Science, University of South Florida, Saint Petersburg, FL, United States of America; 2 Southeast Fisheries Science Center, National Oceanic and Atmospheric Administration, Pascagoula, MS, United States of America

**Keywords:** Fish, Egg, Barcoding, DNA, COI, West Florida Shelf, Early life stage, MOCNESS, Depth

## Abstract

Genetic barcoding of fish eggs has furthered our knowledge of fish spawning patterns and locations, providing valuable insights for conservation and management efforts. Since fish eggs tend to behave as buoyant, passive particles, most studies collect them from surface waters and assume that this method captures eggs from all the species that have recently spawned throughout the water column. To experimentally test this assumption, we used a Multiple Opening/Closing Net and Environmental Sensing System (MOCNESS) to collect fish eggs from six depth bins within the upper 130 m of the water column at five stations on the West Florida Shelf. We used DNA barcoding to identify fish eggs collected within each depth bin to determine if the diversity of eggs recovered was consistent throughout the water column. Fish egg assemblage composition was heterogeneous throughout the water column, with most taxa only detected at one or two distinct depth bins per station, only a few taxa found at more than half the depth bins at any given station, and only a single taxon found at all depths within a single station. Disproving the hypothesis that all eggs present throughout the water column would be detected at the surface, only 19 of the 44 taxa identified in this study were observed in the samples collected from the upper 20 m. These findings suggest that exclusively sampling at the surface provides an incomplete picture of the fish assemblage spawning at a given station, which is difficult to predict due to variability in the rates of egg rise through the water column and further complicated by potential mismatches in the time of spawning relative to when collections are made, encounters with subsurface currents while rising to the surface, and the potential for denser eggs to reach neutral buoyancy at deeper isopycnals.

## Introduction

Understanding fish spawning patterns, including the timing, location, and depth distribution of fish eggs throughout the water column, is vital to the conservation and management of important fish populations. Most marine fishes are broadcast spawners, releasing their eggs directly into the water column, where they hatch within hours to days (Pauly & Pullin, 1988), making them reliable predictors of spawning locations compared to larvae, which develop over days to months and exhibit behavioral features such as active swimming and vertical migration (Cowen & Sponaugle, 2009; Hawes et al., 2020; Stenevik, Sundby & Cloete, 2001). Sustainability of fisheries on the West Florida Shelf (WFS) is a management priority, and several studies have identified fish eggs through genetic barcoding of the mitochondrial cytochrome c oxidase I gene to identify critical habitats needed for successful reproduction in this region (Breitbart et al., 2023; Burghart et al., 2014; Burrows et al., 2019; Keel et al., 2025; Martínez-Mercado et al., 2025). DNA barcoding of fish eggs is more accurate than relying on morphological characterization due to the lack of discerning features that enable discrimination between different taxa (Hofmann et al., 2017; Kawakami, Aoyama & Tsukamoto, 2010).

Although fish can spawn at different depths in the water column, their eggs are passive particles that exhibit positive buoyancy, rising to surface waters post-spawning (Paris & Cowen, 2004). This migration results from egg hydration, and lower salinity levels of the ovoplasm compared to the surrounding seawater (Baras et al., 2018). Therefore, the vast majority of studies collect fish eggs from surface waters, operating under the assumption that broadcast spawned eggs eventually make their way to the surface (Checkley Jr et al., 1997; Tan et al., 2023). This study directly tests the hypothesis that the assemblage of fish eggs collected at the surface reflects the diversity of fish species spawning throughout the water column. We used fish egg samples collected using a Multiple Opening/Closing Net and Environmental Sensing System (MOCNESS) to determine if eggs from different fish species are detected at various depths in the water column on the WFS in February. MOCNESS systems have been successfully deployed for the collection of fish eggs in prior studies and are notably efficient compared to conventional sampling methods, such as bongo nets, catching a higher number of eggs (Johnson & Fogarty, 2013). We acquired samples at six different depth bins (0–20 m, 21–40 m, 41–60 m, 61–80 m, 81–100 m, and 101–130 m) from five stations and performed genetic barcoding to identify individual fish eggs throughout the depth profile. This investigation provides valuable information regarding the composition of fish egg assemblages with respect to depth, yielding important implications for future sampling design and interpretation of results.

## Materials & Methods

### Sample collection

**Figure 1:**
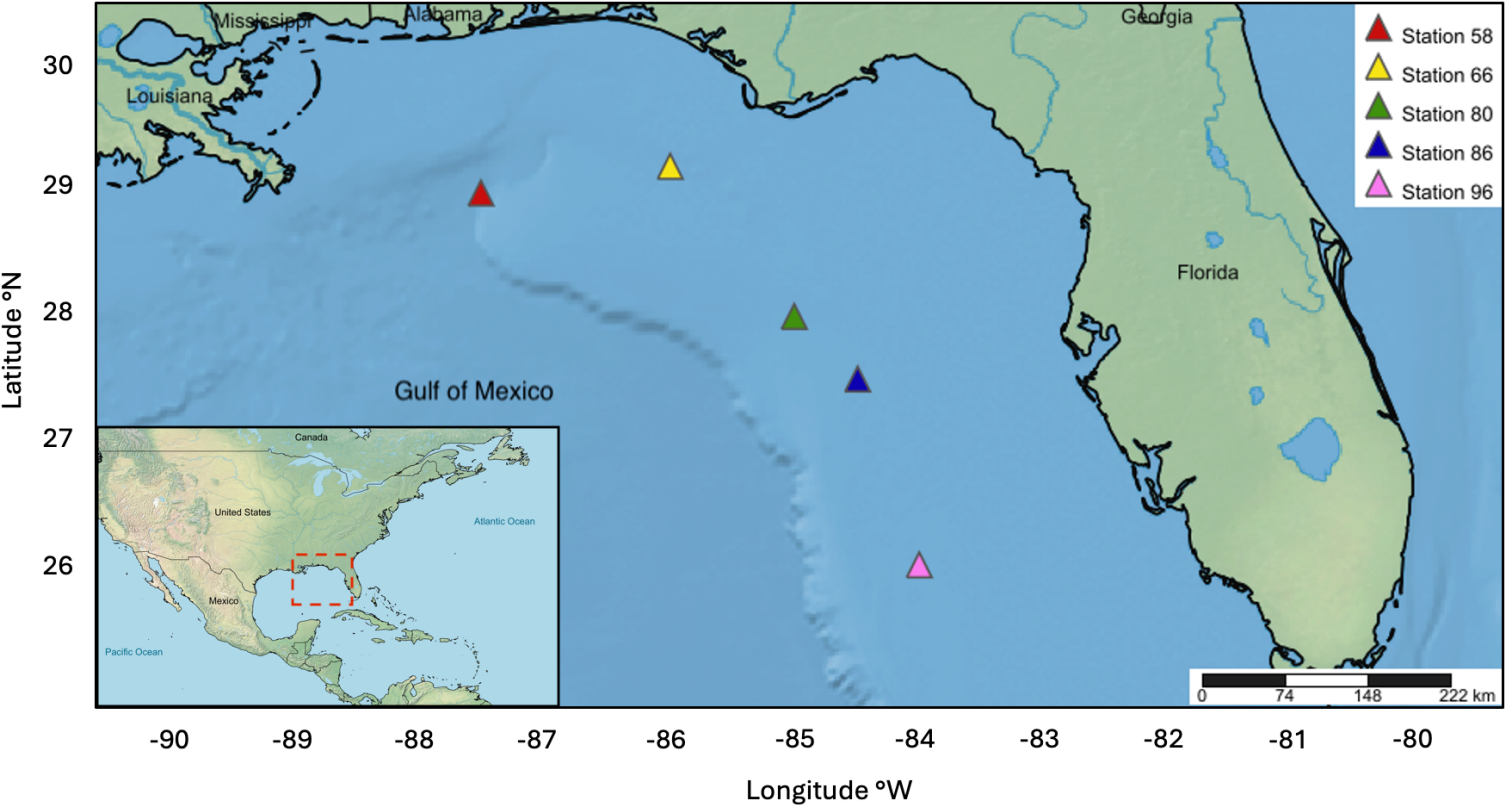
Map displaying five MOCNESS sampling stations within the WFS.

The NOAA Southeast Area Monitoring and Assessment Program (SEAMAP) collected ichthyoplankton samples between February 15-24, 2012 on the WFS as part of a fishery-independent survey aboard the NOAA Ship *Oregon II*. A 1-m^2^ MOCNESS outfitted with 0.505 mm mesh nets was deployed at stations 58 (28° 59′46.8″N 87°30′55.8″W), 66 (29°12′39.0″N, 85°59′46.8″W), 80 (28°00′18.6″N, 85°00′00.6″W), 86 (27° 30′09.0″N, 84°29′28.8″W), and 96 (26°00′36.0″N, 83°59′48.0″W). The nets sampled six discrete depths ranging approximately from: (1) 0–20 m, (2) 21–40 m, (3) 41–60 m, (4) 61–80 m, (5) 81–100 m, and (6) 101–130 m (Fig. 1). Ichthyoplankton samples were initially preserved in 95% ethanol and transferred to fresh 95% ethanol after 24 h. Samples were processed at the Plankton Sorting and Identification Center (ZSIOP) at the Sea Fisheries Institute (MIR) in Poland where zooplankton, fish larvae, and fish eggs were all separated and placed in separate vials. We picked 48 fish eggs per depth bin at each station under a dissecting microscope with tweezers following the pattern of a gridded dish for DNA barcoding analysis. For depth bins containing less than 48 eggs, we picked all available eggs in the same manner.

### DNA extraction & analysis

We performed DNA extractions using the HotSHOT DNA extraction method (Truett et al., 2000) as previously applied to fish eggs (Burrows et al., 2019; Keel et al., 2025; Kerr et al., 2020). We individually transferred fish eggs to 0.2 ml 8-strip polymerase chain reaction (PCR) tubes using a sterile pipette tip and removed any residual ethanol from the tubes by pipetting. We added 50 µl of alkaline lysis buffer containing 0.2 mM disodium EDTA and 25 mM NaOH, pH 12 to each tube, then manually ruptured the eggs using sterile wooden toothpicks. After capping the tubes, we heated them to 95 °C in a thermocycler for 30 min, then cooled on ice for 3 min. Once cooled, we uncapped the tubes and added 50 µl of a neutralization reagent containing 40 mM Tris–HCl (pH 5). Lastly, we recapped all tubes, then vortexed and briefly centrifuged them before storing at −20 °C.

We genetically identified the fish eggs through PCR amplification of the mitochondrial cytochrome c oxidase I (COI) gene using the COI-3 universal fish primer cocktail (Ivanova et al., 2007). Individual PCR reactions of 50 µl contained final concentrations of 1x Apex NH_4_ buffer, 1.5 mM MgCl_2_, 0.2 µM Apex dNTPs, 1 U Apex RedTaq, 0.2 µM COI-3 universal fish primer cocktail, 10 µg/µl bovine serum albumin, and 2 µl of target DNA. We placed PCR samples in a thermocycler and heated to 94 °C for 2 min. This step was followed by 45 cycles of heating and cooling (94 °C for 30 s, 52 °C for 40 s, 72 °C for 1 min), and then a final extension at 72 °C for 10 min. We confirmed the success of PCR amplification through gel electrophoresis of products on a 1.5% agarose gel stained with ethidium bromide. TACGen purified and Sanger sequenced successfully amplified products using the M13 forward primer within the universal fish primer cocktail. We used Geneious software (Kearse et al., 2012) to trim the sequences for quality with the following parameters: maximum of five mismatches, minimum match length of five for primers, and an end error probability limit of 0.05 including trimming at least three base pairs (bp). We compared trimmed sequences longer than 80 bp to species-level identification references within the Barcode of Life Database (BOLD), using a 97% threshold for positive identifications. Data are available in GRIIDC (https://data.griidc.org/data/F2.x836.000:0012) and in Table S2.

## Results

We picked and extracted DNA from a total of 1304 individual fish eggs. We successfully amplified PCR products from 869 eggs (66.6% success rate), obtained high-quality sequences from 737 eggs, and taxonomically identified 623 eggs. As a combined consequence of PCR failure, poor sequence quality, and database limitations, only 47.8% of the eggs processed yielded identifications. We identified 44 distinct fish taxa belonging to 23 families (Table S1). All but three of these identifications could be resolved to the species level with the remainder assigned to the genus level.

**Figure 2:**
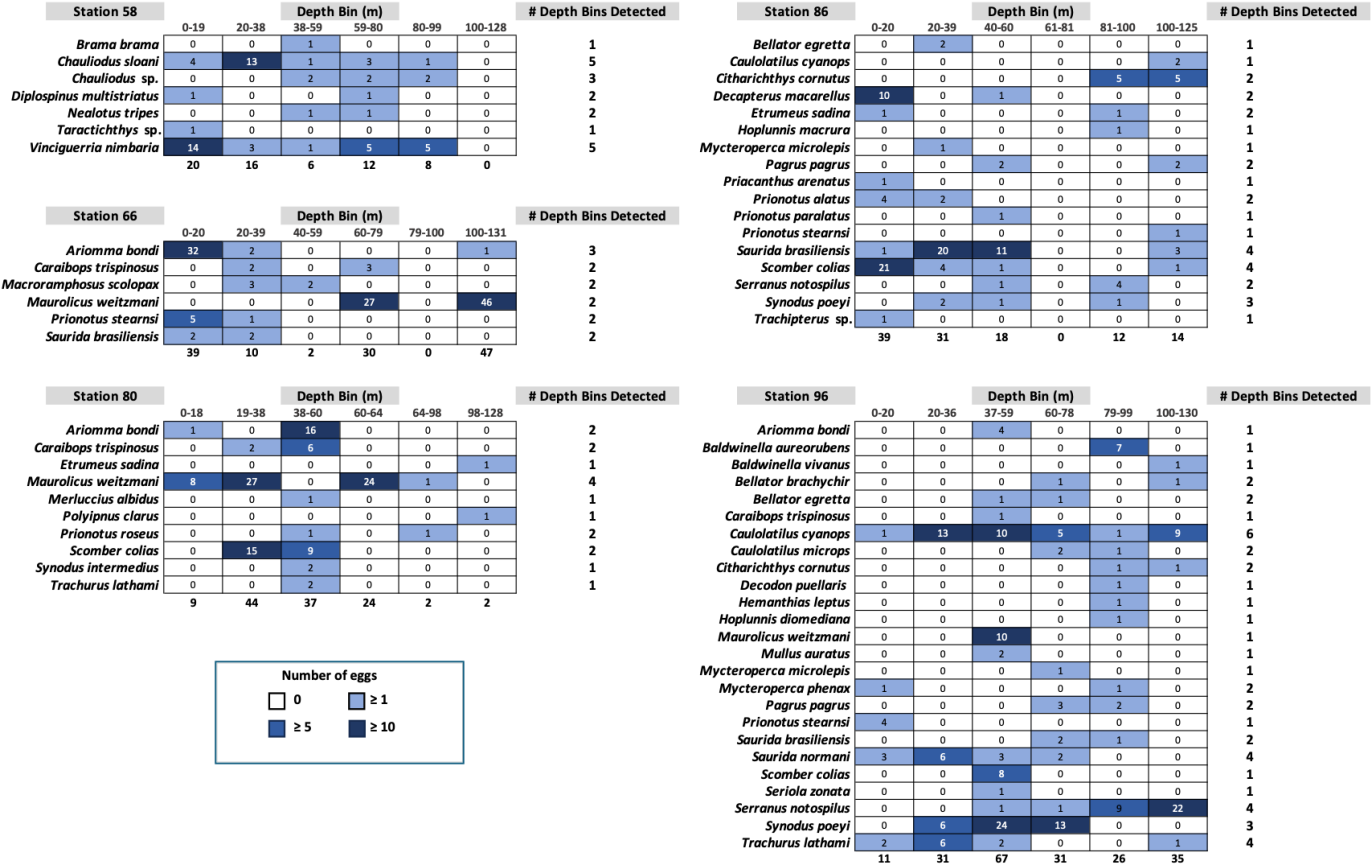
Heatmaps displaying the abundance of fish eggs by species within samples collected from each depth bin at each station.

**Figure 3:**
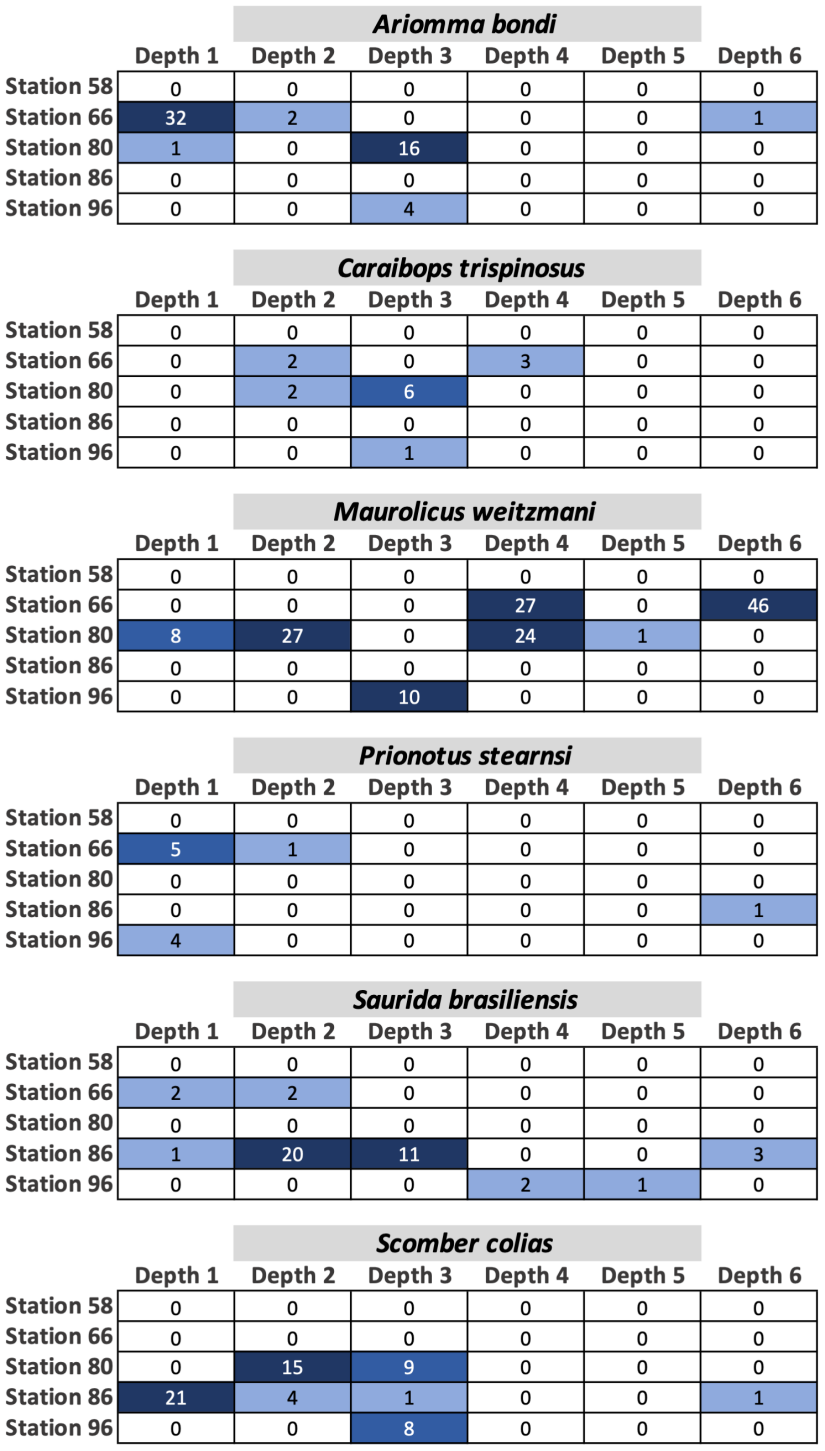
Heatmaps displaying the abundance of fish eggs for species observed at a minimum of three separate stations across varying depths.

Fish egg assemblage composition was highly heterogeneous throughout the water column, with the majority of taxa at any given station only detected at a single (*n* = 28) or two depths (*n* = 24), eight taxa found at more than half the depths, and a single taxon (*Caulolatilus cyanops* at station 96) found at all depths (Fig. 2). Notably, on several occasions a large number of eggs from a given taxon were recovered in a single depth bin, with that taxon either not seen or poorly represented in the surrounding depth bins (*e.g.*, 10 *Maurolicus weitzmani* eggs identified between 37-59 m at Station 96, but none recovered from other depths; Fig. 2). Six taxa were detected at three separate stations (Fig. 3), of these only two, *Maurolicus weitzmani* and *Saurida brasiliensis*, were observed at all six depth bins across three stations.

Contrary to our hypothesis that the egg assemblage recovered from the surface layer would be representative of eggs found throughout the water column, between 20% (stations 80 and 96) and 57% (station 58) of the taxa present at a given station were found in the surface 20 m (Fig. 2). Similarly, 19 of the 44 taxa identified across the five stations in this study (43%) were observed in the samples collected from the upper 20 m (Table S1). The five most abundant taxa across all stations (Table S1) were *Maurolicus weitzmani* (Atlantic pearlside), *Ariomma bondi* (silver-rag driftfish), *Scomber colias* (Atlantic chub mackerel), *Synodus poeyi* (offshore lizardfish), and *Saurida brasiliensis* (Brazilian lizardfish). The combined total eggs from these species accounted for over half (*n* = 347) of the identifications made. Station 96 had the largest number of eggs successfully identified (*n* = 201) and yielded the most distinct identifications with 25 taxa, while station 66 had the second highest number of successfully identified eggs (*n* = 128) yet yielded the lowest number of taxa (*n* = 6).

Of the 44 taxa observed in this study, a majority are demersal (*n* = 16) or reef-associated (*n* = 9) (Table S1). Eggs were observed from species from other habitat categories including bathypelagic, pelagic-neritic, pelagic-oceanic, and benthopelagic. Station 58 contained many bathypelagic species (Fig. 2), these included *Vinciguerria nimbaria* (oceanic lightfish), *Neolotus tripes* (black snake mackerel), and *Chauliodus sp.* (viperfish). Eggs from these species were detected across varying depths. Notable pelagic-neritic fish included *Brama brama* (Atlantic pomfret), *Etumeus sadina* (red-eye round herring), and *Scomber colias* (Atlantic chub mackerel). Pelagic-oceanic fish included *Taractichthys longipinnis* (big-scale pomfret) and *Decapterus macarellus* (mackerel scad).

## Discussion

In contrast to existing literature that has only analyzed fish eggs collected from surface samples, the present study specifically identified fish eggs collected from different depths within the water column using a MOCNESS system, providing the first depth-specific fish egg assemblage composition data to date. The fish egg assemblage was highly heterogeneous, with most species only detected at one or two depths at any given station. Only 40% of the taxa observed throughout the water column were also recovered from the upper 20 m, suggesting that surface sampling is not representative of all spawning happening in deeper waters. Although a large number of the eggs were unidentifiable, primarily due to failure in the PCR amplification step due to the long-term storage of samples before processing, these samples were lost due to hurricane impacts on the lab in 2024 and we are unable to attempt further techniques to increase the success rates. Therefore, we have focused the results and discussion on the eggs that could be identified through DNA barcoding.

**Figure 4:**
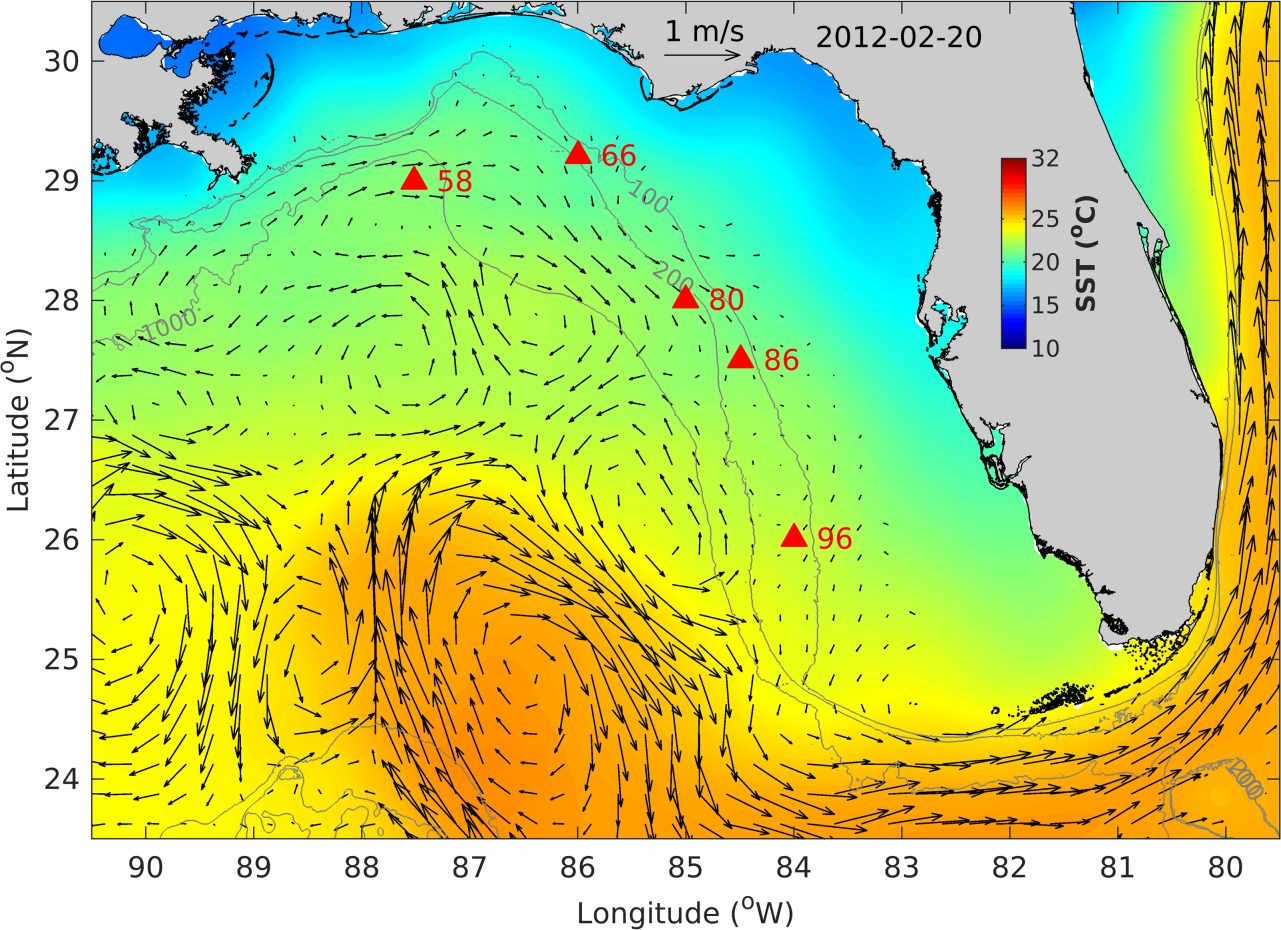
West Florida Shelf map showing the fish egg sampling locations (filled red triangles), altimetry-derived surface currents and sea surface temperature (SST) on 20 February 2012. Also shown are bathymetry contours of 100, 200 and 1,000 m.

**Figure 5:**
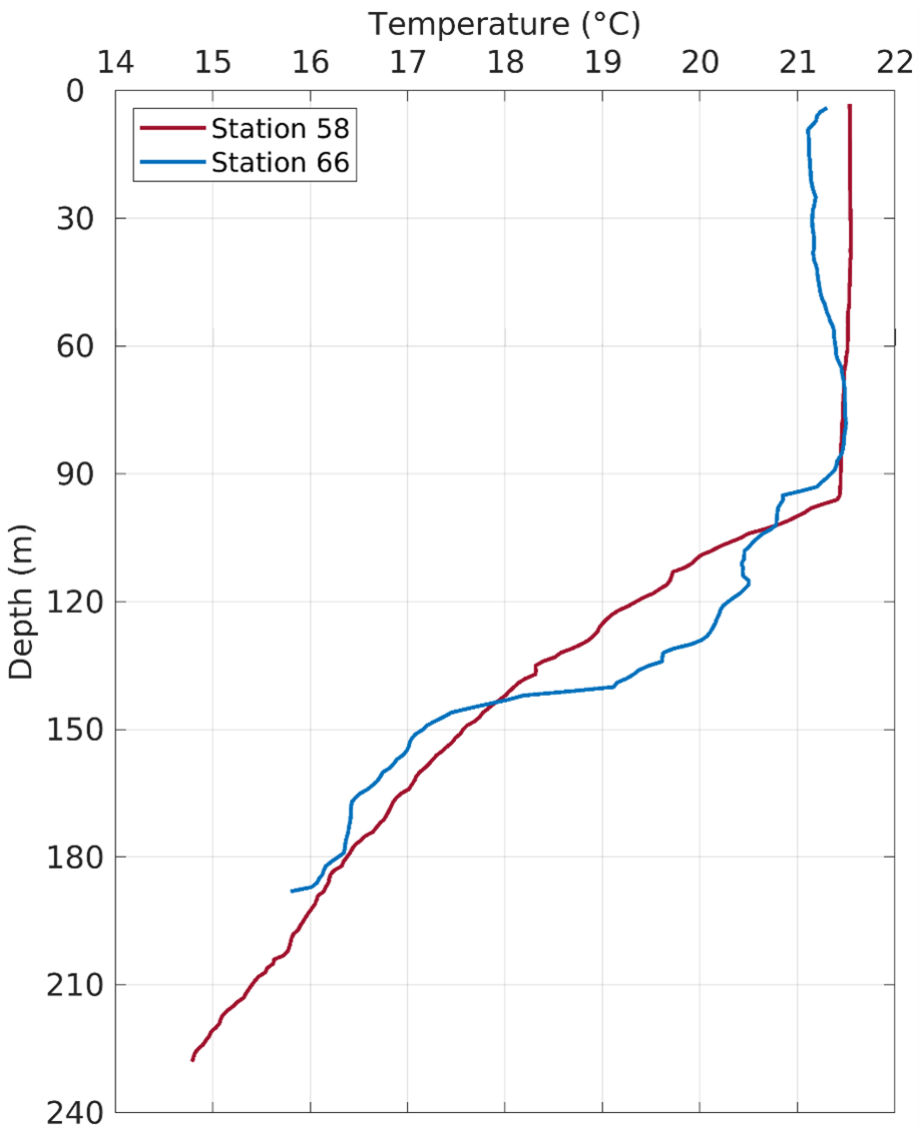
Temperature profiles in the water column at Stations 58 and 66.

Fish eggs are positively buoyant, but may rise at different rates due to variations in egg size, aqueous contents, oil globules, and densities at different stages of development and across fish species (Jung et al., 2014; Ospina-Álvarez, Palomera & Parada, 2012). Eggs may have been sampled during their ascent to the surface, may have reached a neutrally buoyant state at deeper isopycnals, or may have been spawned below a critical depth, in which case they may sink out of the water column (Sundby & Kristiansen, 2015). In addition, turbulent mixing and stratification within the water column also have the potential to affect the migration and settling of fish eggs, potentially delaying their immediate rise to the surface (Conway, 1997). Finally, fish like mackerel and sardines are known to prey heavily upon pelagic fish eggs (Veríssimo, Fonseca & Garrido, 2024); therefore, predation at the surface and adjacent depths may result in depth-related heterogeneity in fish egg assemblages.

Fish egg identification studies using surface-collected eggs frequently employ backward trajectory modeling to predict likely spawning locations (Keel et al., 2025; Nguyen et al., 2024). Hindcast modeling is beneficial for the prediction of horizontal movement since models efficiently capture wind-driven surface currents; however, these methods do not typically consider subsurface currents affecting the fish eggs at various points during their ascent, or the effects of more complicated external factors such as stratification and turbulent mixing on egg distributions. To fine-tune hindcast models of spawning locations, future work should attempt to account for the specific densities of eggs from various fish species, their rates of ascent in given oceanographic regimes, and the currents they are subjected to during ascent to the surface.

The fish egg samples in this study were mostly located on the outer shelf between 100–200 m isobaths, with one station at >1,000 m in the De Soto Canyon area (Fig. 4). West Florida Shelf circulation has been studied extensively using moored observations and numerical models during the last two decades. Its seasonal variation is pronounced on the inner shelf (Liu & Weisberg, 2012), but not obvious on the outer shelf due to the influence of the offshore eddies associated with the Loop Current system (*e.g.*, Liu et al., 2016). To examine the ocean circulation pattern during the study time period, we show a map of satellite altimetry-derived surface geostrophic currents (Fig. 4), mainly because this current product was found to be more accurate than the data assimilative numerical models in this area (Liu et al., 2014). Sea surface temperature (OSTIA product; (Worsfold et al., 2024)) is also shown in Fig. 4. During our study time period, the Loop Current system stayed off from the “Pressure Point” of the shelf (Liu et al., 2016); thus, a strong offshore forced upwelling is not expected for the shelf. Due to CTD malfunction, we only have vertical temperature profiles from stations 58 and 66. These profiles show a mixed layer depth of about 90 –100 m, below which is a weakly stratified lower water column (Fig. 5). The local oceanographic conditions are considered normal for the time of year.

### Conclusion

This study examined fish egg assemblage composition within discrete depth bins from the upper 130 m of the water column on the WFS to test the assumption that surface samples fully represent the fish spawning assemblage at a given location. Our data demonstrate that surface sampling alone does not provide a comprehensive view of all fish eggs present at a given location, with up to 80% of taxa being overlooked, potentially due to factors such as spawning depth, variation in egg buoyancy and ascent rate, density differences throughout the water column, interactions with subsurface currents and turbulence, predation, and the potential for some eggs to reach neutral buoyancy at deeper points within the water column. Results from this study demonstrate the heterogeneity of fish eggs throughout the water column, indicating the advantage of applying depth-specific assessments when characterizing fish spawning assemblages using egg presence. Furthermore, these data will be valuable for future incorporation into hindcasting models to backtrack fish-egg trajectories and pinpoint spawning locations for fish species of interest.

##  Supplemental Information

10.7717/peerj.20498/supp-1 Supplemental Information 1 Detailed information for each fish taxon recovered in this study

10.7717/peerj.20498/supp-2 Supplemental Information 2 Sequences recovered from MOCNESS fish eggs Data from MOCNESS fish egg survey, including sequence_name, sample_name, organism isolation_source, collection_date, collection_time, collection_depth, latitude, longitude, station number, geographic location name, sample collection device description, number of fish eggs, processed sequence

## Additional Information and Declarations

### Data Availability

The following information was supplied regarding data availability: The data are available in the Supplemental File. The data are available at GRIIDC: Arianna Rodriguez, Keith Keel, Glenn Zapfe, Christopher D. Stallings, Mya Breitbart. Data identifying fish eggs obtained from oceanographic cruises at varying depths in West Florida from 2012-02-16 to 2012-02-23 with sequences of the mitochondrial gene. Distributed by: GRIIDC, Harte Research Institute, Texas A&M University–Corpus Christi. https://doi.org/10.7266/hvt73dxz.
